# Anti-Obesity Effect of Kimchi with Starter Cultures in 3T3-L1 Cells

**DOI:** 10.4014/jmb.2307.07005

**Published:** 2023-10-11

**Authors:** In-Kyung Hyun, Sung Wook Hong, Min-Ji Ma, Ji Yoon Chang, Seongsoo Lee, Ye-Rang Yun

**Affiliations:** 1 World Institute of Kimchi, Nam-Gu, Gwangju 61755, Republic of Korea; 2 Gwangju Center, Korea Basic Science Institute (KBSI), Gwangju 61751, Republic of Korea

**Keywords:** Anti-obesity, Kimchi, lactic acid bacteria, starter, triglyceride, 3T3-L1 cells

## Abstract

Lactic acid bacteria (LAB) isolated from kimchi have various functions, including antioxidant, anti-inflammation, and anti-obesity activities, and are therefore widely used in the food, pharmaceutical, and medical fields. To date, the health functionalities of LAB have been widely reported; however, those of kimchi fermented with LAB as a starter have rarely been reported. Therefore, research on the selection of LAB with anti-obesity activity and the health functionality of kimchi fermented with LAB is needed. In the present study, LAB with anti-obesity activity were initially selected by measuring the Oil-Red O intensity. Among the four LAB strains, anti-obesity activity was confirmed by measuring cell viability, lipid levels, and lipid accumulation. Then, starter kimchi (SK) was prepared by inoculating selected LABs, and its pH, total acidity, and salinity were compared with those of naturally fermented kimchi (NK). Lastly, anti-obesity activity was also investigated in 3T3-L1 cells. Selected LAB showed no cytotoxicity up to 10^7^ CFU/ml, with *Lactobacillus brevis* JC7 and *Leuconostoc mesenteroides* KCKM0828 having higher inhibitory effects on TG, TC content and lipid accumulation. Most SKs showed fermentation properties similar to those of the NK. SKs showed no cytotoxicity at concentrations of up to 1,000 μg/ml. SKs showed strong inhibitory effects on TG content, lipid accumulation, and obesity-related gene and protein expressions. Taken together, the utilization of LAB as a starter could improve the health benefits of kimchi.

## Introduction

Lactic acid bacteria (LAB) colonize the host intestine and participate in various physiological and metabolic processes by producing metabolites [1]. LAB are commonly isolated from numerous food sources, including kimchi, soybean paste, chili pepper paste, and milk. Interest in isolated LAB is growing worldwide owing to their health benefits, which have been demonstrated in cells, animals, and humans [2-4]. For instance, the combination of *Lactobacillus plantarum* and tacrolimus monotherapy improves colitis induced by dextran sulfate sodium in mice [5]. Similarly, *Lactobacillus johnsonii* alleviates colitis by suppressing inflammation [6]. Along with improvements in intestinal health, an anti-obesity effect has also been demonstrated [7-11]. *L. plantarum* KU15117, isolated from kimchi, reduces triglyceride (TG) content, lipid accumulation, and adipocyte differentiation-related gene expression levels [7]. According to a study by Ban *et al*., *Bifidobacterium lactis* IDCC4301 demonstrates anti-obesity effects in differentiated cells and obese mice, which shows promise as a potential treatment for obesity [10]. In a clinical study, *Limosilactobacillus fermentum* strains MG4231 and MG4244 have shown anti-obesity effects by reducing body weight and fat in overweight and obese individuals [11]. Hence, manufacturers are also producing functional food products containing LAB to exploit these benefits. Furthermore, interest in LAB is increasing with regard to pharmaceutical and medical applications.

Studies have also been conducted on the health functionality of foods using LAB as starter cultures [12-15]. Gu *et al*. revealed that *Lactobacillus plantarum* dy-1-fermented barley extract induces the browning of 3T3-L1 adipocytes and reduces body weight and lipid profiles in diet-induced obese mice [12]. Soymilk fermented with *Leuconostoc kimchii*, *Leuconostoc citreum*, and *L. plantarum* isolated from kimchi shows anti-obesity effects in cells and mice [14]. In addition, studies have demonstrated various characteristics of LAB in kimchi fermentation, with potential implications for their use as kimchi starter cultures, as well as the health benefits of LAB in kimchi fermentation [16-19]. According to a study by Seo *et al*. [18], *Limosilactobacillus fermentum* and *Limosilactobacillus reuteri* show similar growth rates, metabolites, and sensory test results as those of *Leuconostoc mesenteroides* DRC 1506, suggesting their potential as kimchi starter cultures. In addition, *L. reuteri* EFEL6901 exhibits anti-inflammatory effects in vitro and in vivo, potentially enhancing the health benefits of kimchi [19]. LAB produce various metabolites and improve the quality and functionalities of kimchi [20-24]. Organic acids, free sugars, and amino acids are the major metabolites produced during kimchi fermentation which contribute to its quality. These metabolites vary considerably depending on the LAB as well as the kimchi ingredients, including salt and starch sauce [20-22]. Vegetable juice fermented using LAB is known to produce indole-3-lactic acid, leucic acid, and phenyllactic acid as metabolites, which inhibit lipid accumulation in vitro [23]. Similarly, exopolysaccharides play pivotal roles in fermented dairy products and in human health [24]. Kimchi fermentation patterns and health functionalities have been reported in studies using LAB as kimchi starter cultures [25-27]. Kimchi fermented using LAB starters (*L. mesenteroides* and *L. plantarum*) shows anti-obesity effects by reducing the lipid content and obesity-related gene levels in 3T3-L1 cells [26]. Although the health benefits of kimchi are known, further research is needed on the functionality of kimchi prepared using LAB as a starter culture, warranting continued investigation in this area.

This study aimed to investigate the anti-obesity activity of starter kimchi (SK) in 3T3-L1 cells. Initially, LAB with anti-obesity activity were selected, and their effects were compared by measuring cell viability, TG content, TC content, and lipid accumulation in 3T3-L1 cells. SKs were prepared by inoculating the selected LAB, and their fermentation properties (pH, total acidity, and salinity) were investigated. The anti-obesity activities of freeze-dried SKs were investigated in 3T3-L1 cells by measuring cell viability, TG content, lipid accumulation, and obesity-related gene/protein expressions.

## Materials and Methods

### Chemicals

The cell counting assay was performed using a Cell Counting Kit-8 (CCK-8) (Dojindo Laboratories Co., Ltd., Japan). TRIzol reagent was purchased from Invitrogen (Carlsbad, USA). TOPScript cDNA Synthesis Kit and SYBR Green premix were purchased from Enzynomics Inc. (Republic of Korea).

### Bacterial Strains

To select LAB for application as kimchi starters, 50 LAB were screened by measuring lipid accumulation. LAB were directly isolated from kimchi or obtained from the Bank of Kimchi Resources and Information. The collected LAB consisted of 4 bacterial strains, including 21 *L. mesenteroides*, one *Lactobacillus brevis*, nine *L. plantarum*, and 19 *Wissela koreensis*. LAB were grown in deMan, Rogosa and Sharpe (MRS) medium (BD Difco, USA). LAB were collected after centrifugation (6,000 ×*g*, 10 min, 4°C) and washed twice with phosphate-buffered saline (PBS).

### Selected Bacterial Strains

In the LAB screening test, two LAB strains that showed anti-obesity effects in a previous study [23] were selected. The selected LAB strains were grown in the same manner as above. The anti-obesity effects of LAB were then evaluated. Information on the selected LAB strains from kimchi is presented in Table 1.

### Preparation of SKs

JC7, KCKM0828, WiKim39, and WiKim0124 cells were cultured in MRS medium to prepare the SKs. The kimchi was composed of ingredients including brined kimchi cabbage, red pepper, garlic, ginger, onions, and radishes. SK1, SK2, SK3, and SK4 cells were inoculated with kimchi starters JC7, KCKM0828, WiKim39, and WiKim0124 (10^7^ CFU/ml). Naturally fermented kimchi (NK) prepared without a kimchi starter was used as a control. Kimchi was stored at 6°C for 4 weeks, and the fermentation properties were measured at 0, 1, 2, and 4 weeks. All kimchi samples were fermented to pH 4.2 and freeze-dried for further analysis.

### pH, Total Acidity, and Salinity of SKs

SKs were blended to measure their fermentation properties. The pH and total acidity were measured using a pH meter (TitroLine 5000, SI Analytics GmbH, Germany). The salinity of the diluted SKs was titrated against 0.02 N AgNO_3_ until a red-brown color changed after the addition of a 2% potassium chromate solution.

### Cell Culture

3T3-L1 cells were obtained from the American Type Culture Collection (ATCC, USA) and cultured in Dulbecco’s Modified Eagle Medium (DMEM) (10% fetal calf serum and 1% penicillin/streptomycin). In the differentiation experiment, cells were differentiated with DMEM (10% fetal bovine serum, 0.5 mM 3-Isobutyl^-1^-methylxanthine, 1 μM dexamethasone, and 5 μg/ml insulin) for 3 days. The cells were maintained in DMEM (10%fetal bovine serum and 5 g/ml insulin) for 8 days.

### Cell Viability Analysis

The cell viability effects of LAB and kimchi were examined using a CCK-8 kit. The 3T3-L1 cells (1 × 10^4^ cells/well) were seeded and incubated with LAB or SKs for 1 day. In the LAB study, each strain was used at 10^5^, 10^6^, 10^7^, and 10^8^ CFU/ml, and MRS medium was used as the control. In the kimchi study, SKs were used at various concentrations (50, 100, 250, 500, 1000, and 2500 μg/ml), and kimchi without a starter was used as the control. The cells were washed three times with PBS and incubated with 20 μl CCK-8 solution for 2 h. The absorbance was measured at 450 nm.

### TG and Total Cholesterol (TC) Content Analysis

The lipids of the 3T3-L1 cells were extracted using a solvent mixture (700 μl, chloroform/methanol/H_2_O mixture, 8:4:3, v/v/v), as previously described [28]. Extracted lipids from the cells were incubated at room temperature (24–26°C) for 60 min, and the organic layer was obtained by centrifugation at 800 ×*g* for 10 min and dried. Dried lipids were dissolved in ethanol (20 μl) and the TG and TC contents were measured (Asan Pharmaceutical, Republic of Korea).

### Oil Red O (ORO) Saining

Differentiated 3T3-L1 cells were washed and fixed in 10% formalin for 30 min. The cells were stained with ORO solution for 15 min at room temperature (24–26°C) and washed. For lipid quantification, stained cells were extracted with 4% NP-40, and the absorbance was measured at 510 nm.

### Quantitative Real-Time PCR (qPCR)

Total RNA was extracted using TRIzol reagent, and cDNA was synthesized using the cDNA synthesis kit. qPCR of cDNA was conducted using SYBR Green Premix and primers (Table 2). The qPCR conditions were as follows: activation at 94°C for 10 min, denaturation for 45 cycles at 94°C for 15 s, and annealing and extension at 60°C for 1 min. qPCR results were normalized to those of *GAPDH*.

### Western blot Analysis

Differentiated 3T3-L1 cells were lysed with PRO-PREP Protein Extraction Solution (iNtRON, Korea) in ice for 1h, followed by centrifugation at 10,000 ×*g* for 10 min at 4°C. Proteins (30 μg) were subjected to Tris-Glycine gel electrophoresis and then transferred to polyvinylidene difluoride membranes (Bio-Rad, USA). The membranes were incubated with primary antibodies including *GAPDH*, C/EBPα, PPARγ, and SREBP-1c (1:1000, Cell Signaling Technology, USA), followed by incubation with anti-rabbit secondary antibodies (Cell Signaling Technology). Protein bands were visualized using an enhanced chemiluminescence system (ECL Advance, GE Healthcare, UK). Protein density was calculated using the Image J 1.53 program (NIH, USA).

### Statistical Analyses

Data are expressed as mean ± standard deviation. Significant differences were evaluated by one-way analysis of variance, and Duncan’s multiple range test using GraphPad Prism 7 (GraphPad, Inc., USA). Statistical significance was set at *p* < 0.05.

## Results and Discussion

### Selection of LAB for Kimchi Application

**Fig. 1:**
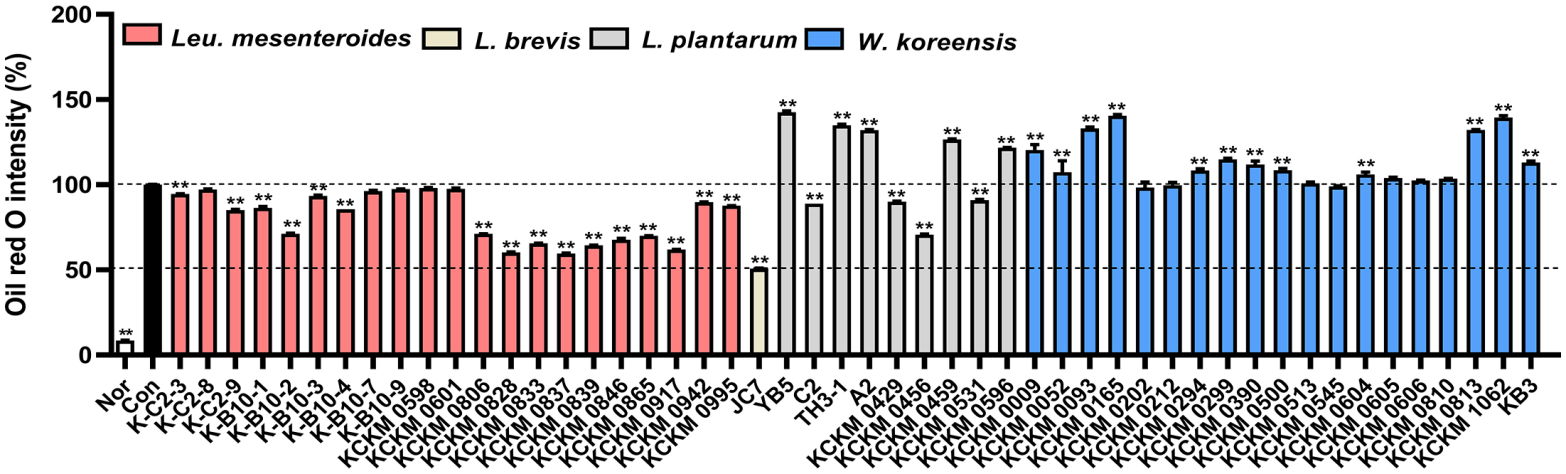
Screening test for the selection of lactic acid bacteria (LAB). Differentiated 3T3-L1 cells were fixed, stained with Oil red O (ORO), and quantified. Results are denoted as mean ± standard deviation (SD). **p < 0.01, vs. Con (differentiated cells without LAB).

We identified the following four LAB strains: *L. brevis* JC, *L. mesenteroides* KCKM0828, *Companilactobacillus allii* WiKim39 [29], and *Lactococcus lactis* WiKim0124. JC7 and KCKM0828 showed powerful inhibitory effects on the ORO intensity test among 50 LAB isolated from kimchi (Fig. 1). The anti-obesity effects of WiKim39 and WiKim0124 have been confirmed earlier in cells and animals. Subsequently, the anti-obesity effects were compared among the four selected LAB strains.

### Effect of LAB on Cell Viability

**Fig. 2:**
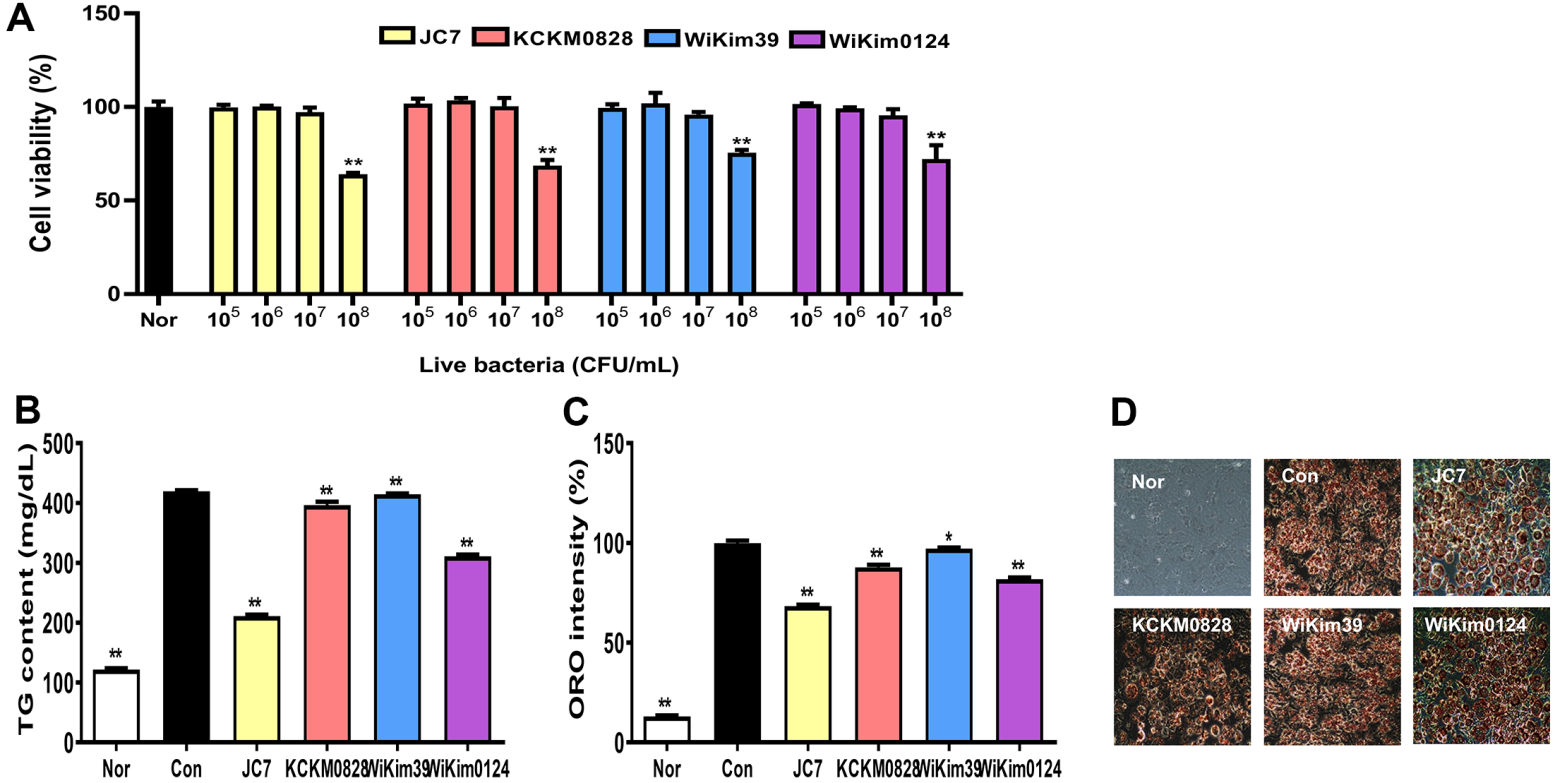
Cell viability, triglyceride (TG) content, and lipid accumulation of LAB in 3T3-L1 cells. Cell viability **A**, TG content **B**, ORO intensity **C**, and ORO image **D**. 3T3-L1 cells were incubated with LAB (10^5^, 10^6^, 10^7^, and 10^8^ CFU/ml) for 24 h. Cytotoxicity was measured using a CCK-8 kit. Lipids of differentiated 3T3-L1 cells were extracted, and the TG content was measured using a kit. Differentiated 3T3-L1 cells were fixed, stained with ORO, and quantified. Results are expressed as mean ± SD. **p < 0.01, vs. Nor (De Man–Rogosa–Sharpe media without LAB). *p < 0.05 and **p < 0.01, vs. Con (differentiated cells without LAB).

Viabilities of the four LAB strains were investigated on 3T3-LI cells. Fig. 2A shows that cell viabilities were approximately 100% up to 10^7^ CFU/ml for all the LAB strains. However, these were reduced to less than 80% at 10^8^ CFU/ml and the viability of cells inoculated with JC7 at 10^8^ CFU/ml was approximately 65%. Typically, the viability effects of LAB vary depending on the cell type (live, heat-killed, and lysed cells). Six heat-killed LAB strains (10^8^ CFU/ml) have shown no cytotoxicity in a previous study [30]. *L. plantarum* HAC01 and *L. plantarum* ATG-K2 show no cytotoxicity in LAB lysate studies up to 400 μg/ml [31, 32]. Based on our cell viability results, a concentration of 10^7^ CFU/ml was used in subsequent experiments.

### Effect of LAB on TG Content and Lipid Accumulation

To determine the effect of LAB (10^7^ CFU/ml) on lipid accumulation inhibition, the TG content was measured. Fig. 2B shows that the TG content of cells inoculated with LAB was reduced, except for that of cells inoculated with WiKim39. Among the LAB strains, JC7 had the greatest inhibitory effect on TG content, followed by KCKM0828, WiKim0124, and WiKim39 in that order. Similar to our results, the six heat-killed strains showed different inhibitory effects on TG content [30]. In contrast to the weak inhibitory effect observed in this study, WiKim39 and WiKim0124 are known to decrease TG content and inhibit lipid accumulation, both in vitro and in vivo [23].

As shown in Figs. 2C and 2D, JC7 had the lowest number of stained cells, which is consistent with the TG content results. The order of the inhibitory effects on lipid accumulation was as follows (greatest effect first): JC7, KCKM0828, WiKim0124, and WiKim39, consistent with the TG results. Taken together, JC7 and KCKM0828 showed strong inhibitory effects on TG content and lipid accumulation, suggesting their potential use as kimchi starters.

### Effect of SKs on Kimchi Fermentation Properties

The fermentation properties (pH, total acidity, and salinity) of the SKs were investigated. Table 3 shows that the pH levels of SK1, SK2, and SK4 were similar to those of the control (NK); however, the pH level of SK3 drastically decreased at 4 weeks. The total acidity of SKs steadily increased during kimchi fermentation (Table 3). At 1 and 2 weeks, the total acidity of SK1 and SK2 increased slowly. SK3 had the lowest salinity among all the kimchi groups (Table 3). To use LAB as a kimchi starter, it is important to determine whether kimchi with a starter exhibits general kimchi fermentation properties. Kimchi fermentation properties differ depending on LAB type and inoculation [8]. In a previous study, the use of a complex starter extended the shelf-life and enhanced sensory qualities more efficiently than those by single starter, suggesting improved kimchi quality [33]. In this study, the addition of a kimchi starter resulted in kimchi fermentation properties similar to those of kimchi without a starter, increasing the possibility of its utilization as a starter in future experiments.

### Effect of SKs on Cell Viability

**Fig. 3:**
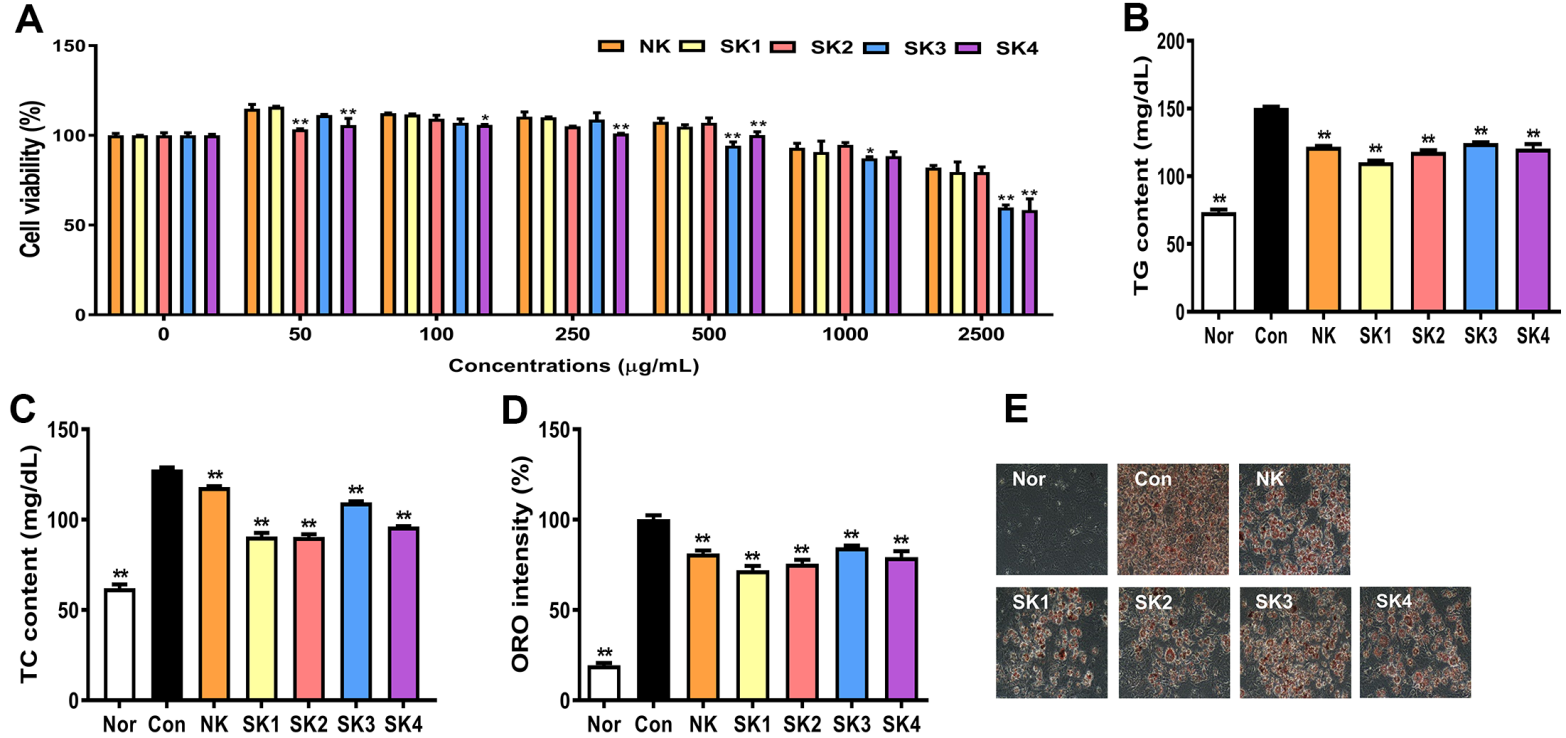
Cell viability, lipid content, and lipid accumulation of SKs in 3T3-L1 cells. Cell viability **A**, TG content **B**, total cholesterol (TC) content **C**, ORO intensity **D**, and ORO image **E**. 3T3-L1 cells were incubated with kimchi (50, 100, 250, 500, 1000, and 2500 μg/ml) for 24 h. Cytotoxicity was measured using a CCK-8 kit. Lipids of differentiated 3T3-L1 cells were extracted, and the TG and TC contents were measured using a kit. Differentiated 3T3-L1 cells were fixed, stained with ORO, and quantified. Results are expressed as mean ± SD. *p < 0.05 and **p < 0.01, vs. NK (kimchi without LAB). **p < 0.01, vs. Con (differentiated cells without LAB).

Fig. 3A shows the cell viability of SKs at various concentrations. Cell viability was approximately 80% up to 1,000 μg/ml for all kimchi groups. However, the cell viability of SK3 and SK4 at 2,500 μg/ml significantly decreased to 60%. According to previous results, the viability of cells inoculated with kimchi differed depending on the kimchi type and recipe. In our previous studies, kimchi with citrus concentrate showed over 80% cell viability upto 500 μg/ml in 3T3-L1 cells [28], while solar salt-brined kimchi showed around 90% cell viability upto 2,500 μg/ml in RAW264.7 cells [34]. Based on the cell viability results, SKs at 1,000 μg/ml were used in subsequent experiments.

### Effect of SKs on Lipid Profiles and Lipid Accumulation

As shown in Fig. 3B and 3C, SKs reduced TG and TC content, which was increased by differentiation. The TG and TC contents of the SK1 group were lowest among all kimchi groups. The inhibitory effects on TC and TG content were in the following order: SK1, SK2, SK4, and SK3. Based on the findings of our previous study [28], TG and TC contents decrease even with normal kimchi; therefore, the TC and TG content reduction of kimchi with LAB as a starter was expected.

Similarly, the number of stained cells in the SK1 group was the lowest, which was consistent with the TG content results (Fig. 3D and 3E). The order of inhibitory effects on lipid accumulation was as follows: SK1 > SK2 > SK4 > SK3. One study demonstrated that NK, FK (NK fermented with green tea), and FKS (FK fermented with a starter) show fewer and smaller lipid droplets than those in the controls [26]. Consistent with the LAB results, SK1 and SK2 showed strong inhibitory effects on TG content and lipid accumulation.

### Effect of SKs on Obesity-Related Gene Levels

**Fig. 4:**
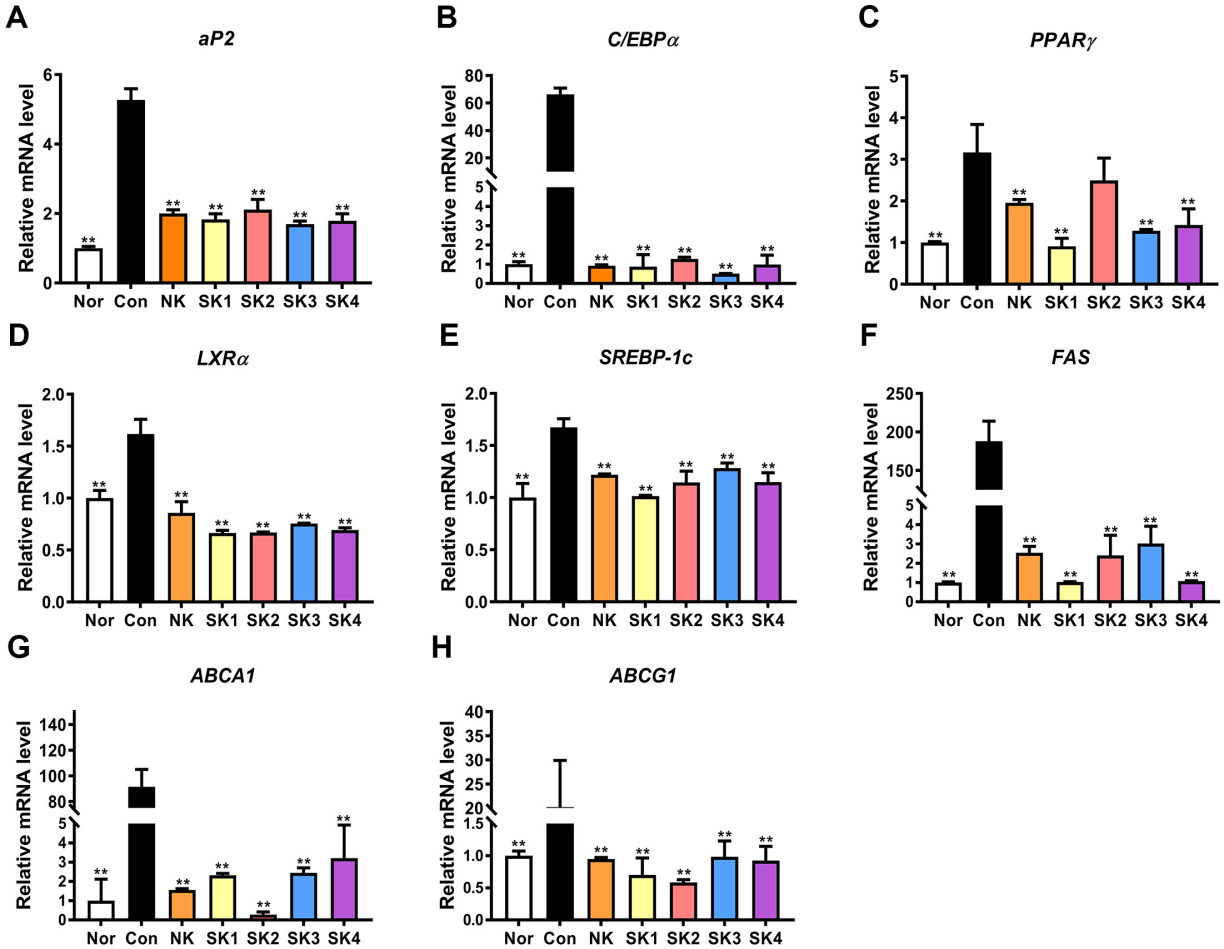
Obesity-related gene expression of SKs in 3T3-L1 cells. *aP2* **A**, *C/EBPα* **B**, *PPARγ* **C**, *LXRα* **D**, *SREBP-1c* **E**, *FAS* **F**, *ABCA1* **G**, and *ABCG1* **H**. Total RNA of differentiated 3T3-L1 cells was extracted and cDNA was synthesized, after which quantitative real-time PCR was performed. Results are expressed as mean ± SD. *p < 0.05 and **p < 0.01, vs. Con (differentiated cells without LAB).

To confirm the anti-obesity effects of SKs, obesity-related genes, including adipogenic, lipogenic, and cholesterol efflux genes, were investigated. Three transcription factors—adipocyte fatty acid-binding protein (*aP2*), CCAAT/enhancer-binding protein α (*C/EBPα*), and peroxisome proliferator-activated receptor γ (*PPARγ*)— induce the expression of genes related to adipogenic differentiation [35]. Fig. 4A–4C shows that the adipogenic gene levels in SKs were significantly reduced compared with those in the controls (*p* < 0.05). Although the inhibitory effects of SKs differed depending on the adipogenic gene, SK1 and SK3 had stronger inhibitory effects. In a previous study, FKS significantly downregulated the gene expression of *PPARγ* and *C/EBPα* [26].

Next, the lipogenic gene levels of SKs were significantly reduced compared with those in the controls, as shown in Fig. 4D–4F (*p* < 0.05). SK1 showed the strongest inhibitory effect on lipogenic gene expression. LXRα, sterol regulatory element-binding protein (*SREBP-1c*), and fatty acid synthase (*FAS*) participate in lipogenic differentiation [36]. The aforementioned study supports our results, showing reduced FAS gene expression [26]. These results confirm the anti-obesity effect of SKs by controlling adipogenic and lipogenic gene levels in 3T3-L1 cells.

ATP-binding cassette A1 (*ABCA1*) and ATP-binding cassette G1 (*ABCG1*) participate in cholesterol efflux, leading to the control of lipid metabolism, and are regulated by *LXR-α* [37]. Figs. 4G and 4H show that the gene levels of *ABCA1* and *ABCG1* were significantly reduced in NK and SKs (*p* < 0.05). Interestingly, this effect was highest with the SK2 treatment, similar to the *LXR-α* results. Based on these results, SKs using LAB exhibit anti-obesity effects through the regulation of obesity-related gene expression levels.

### Effect of SKs on Obesity-Related Protein Levels

**Fig. 5:**
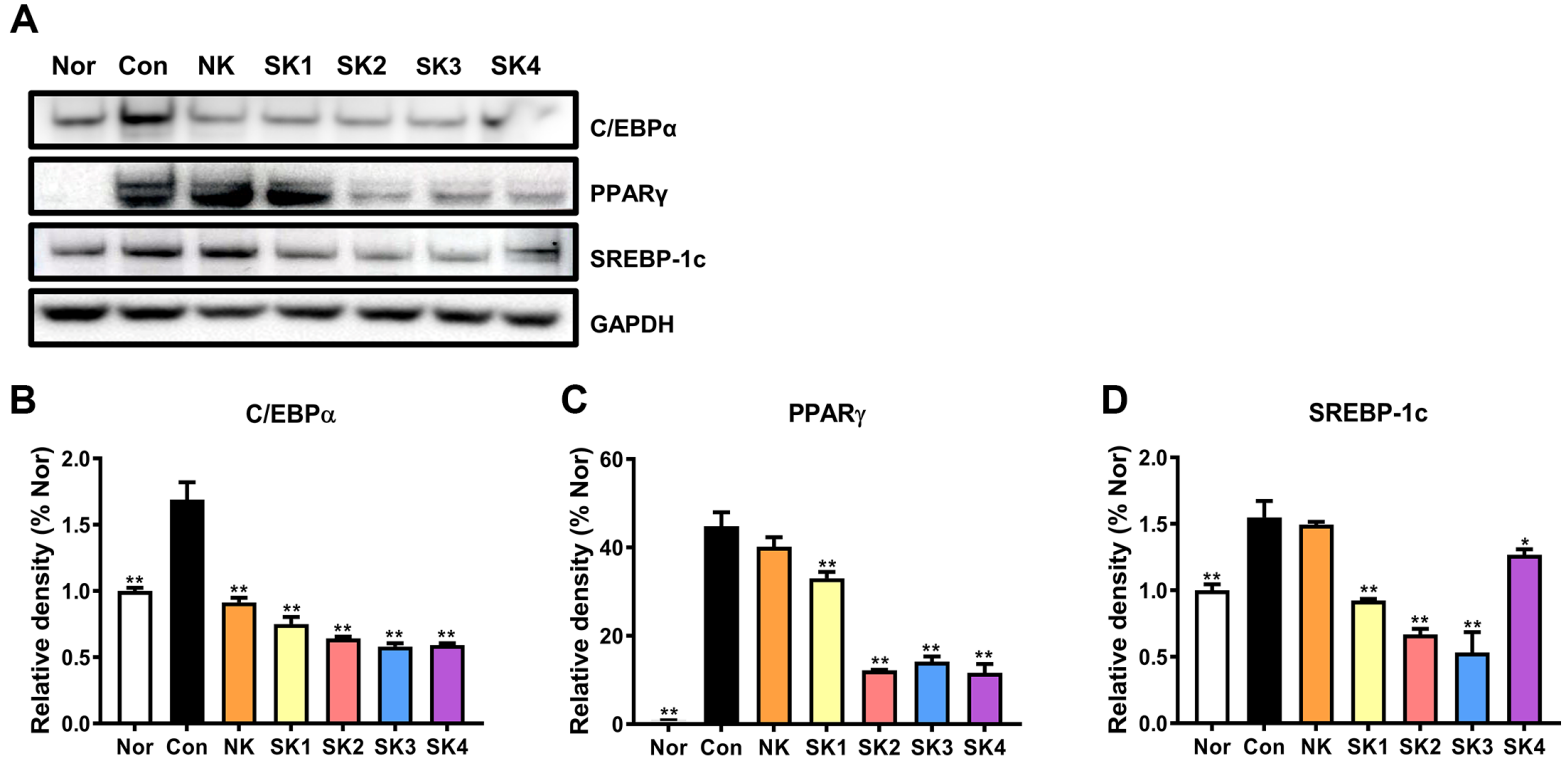
Obesity-related protein expression of SKs in 3T3-L1 cells. Obesity-related protein expression **A**, relative density of C/EBPα **B**, relative density of PPARγ **C**, relative density of SREBP-1c **D**. Proteins from differentiated 3T3-L1 cells were extracted and analyzed using western blot analysis. Results are expressed as mean ± SD. *p < 0.05 and **p < 0.01, vs. Con (differentiated cells without kimchi).

Along with obesity-related gene levels, we investigated the effect of kimchi on obesity-related protein levels using western blotting. Fig. 5 shows the alteration of C/EBPα, PPARγ, and SREBP-1c protein levels. Consistent with the gene results, NK and SKs reduced the levels of all the three proteins. Particularly, SKS significantly reduced the protein levels (*p* < 0.05). Although not related to kimchi, the findings of previous studies are consistent with the results of this study [38, 39]. Heat killed EF-2001dose-dependently reduces C/EBPα and PPARγ, protein expression levels [38]. In addition, quinzarin, a natural phenolic compound, also dose-dependently reduces the expression of these proteins [39]. Thus, SKs exhibit anti-obesity effects by suppressing obesity-related protein as well as gene expression levels.

This study had some limitations. First, even though the fermentation properties of SKs are similar to those of NK, we did not conduct an informal sensory evaluation. Second, although we revealed the anti-obesity effects of SKs in cellular systems, further studies are needed to confirm their effects in animal systems. Thus, we are currently conducting an animal study to evaluate these findings in vivo. Lastly, research on the anti-obesity functional mechanism of kimchi with LAB as starter is required in future.

In conclusion, this study demonstrated the anti-obesity effects of the four selected LAB strains on 3T3-L1 adipocytes. In addition, the anti-obesity effects of SKs were confirmed by measuring the TG content, TC content, lipid accumulation, and obesity-related gene/protein levels. In summary, the health functionality of kimchi can be improved using appropriate LAB as starters. Additionally, these anti-obesity effects of SKs might be attributed to active components and metabolites as well as LAB themselves. In future studies, the anti-obesity effects of the selected SKs need to be verified in animal models and the associated mechanisms need to be evaluated.

## Figures and Tables

**Table 1:** LAB from kimchi used in this study.

LAB	Strains	Kimchi source
*JC7*	*Lactobacillus brevis* JC7	Radish kimchi
*KCKM0828*	*Leuconostoc mesenteroides* KCKM0828	Cabbage kimchi
*WiKim39*	*Companilactobacillus allii* WiKim39	Scallion kimchi
*WiKim0124*	*Lactococcus lactis* WiKim0124	Cabbage kimchi

**Table 2:** Primer sequences for quantitative real-time PCR.

Gene	Forward primer (5'-3')	Reverse primer (5'-3')
*GAPDH*	GTATGACTCCACTCACGGCAAA	GGTGTGGCTCCTGGAAGATG
*aP2*	CATGGCCAAGCCCAACAT	CGCCCAGTTTGAAGGAAATC
*C/EBPα*	AGGTGCTGGAGTTGACCAGT	CAGCCTAGAGATCCAGCGAC
*PPARγ*	TGGAATTAGATGACAGCGACTTGG	CTGGAGCAGCTTGGCAAACA
*LXRα*	CTCAATGCCTGATGTTTCTCCT	TCCAACCCTATCCCTAAAGCAA
*SREBP-1c*	AGAGGGTGAGCCTGACAA	CCTCTGCAATTTCCAGAT
*FAS*	TCTGAGCAGGTGCAGGAGGA	GTTGTTCCTCCAGTTCCGATTTGTA
*ABCA1*	GGTTTGGAGATGGTTATACAATAGTTGT	TTCCCGGAAACGCAAGTC
*ABCG1*	AGGTCTCAGCCTTCTAAAGTTCCTC	TCTCTCGAAGTGAATGAAATTTATCG

**Table 3:** pH, total acidity, and salinity of SKs during kimchi fermentation.

Week	Samples	pH	Total acidity (%)	Salinity (%)
0	NK	5.75 ± 0.01	0.45 ± 0.00	2.01 ± 0.01
	SK1	5.73 ± 0.01	0.44 ± 0.00	1.97 ± 0.01**
	SK2	5.74 ± 0.01	0.45 ± 0.00	2.11 ± 0.00**
	SK3	5.75 ± 0.01	0.43 ± 0.00	2.03 ± 0.01**
	SK4	5.76 ± 0.01*	0.43 ± 0.00	1.86 ± 0.01**
1	NK	4.37 ± 0.01	1.02 ± 0.01	2.05 ± 0.02
	SK1	4.58 ± 0.01**	0.74 ± 0.00**	1.97 ± 0.01**
	SK2	4.68 ± 0.01**	0.71 ± 0.00**	2.06 ± 0.00**
	SK3	4.43 ± 0.01**	0.85 ± 0.00**	1.99 ± 0.01
	SK4	4.65 ± 0.01**	0.69 ± 0.00**	1.97 ± 0.01
2	NK	4.15 ± 0.02	1.13 ± 0.00	2.02 ± 0.01
	SK1	4.31 ± 0.01**	1.03 ± 0.00**	1.88 ± 0.01**
	SK2	4.36 ± 0.02**	1.01 ± 0.00*	2.05 ± 0.01**
	SK3	4.21 ± 0.01**	1.11 ± 0.01**	2.00 ± 0.01**
	SK4	4.24 ± 0.01**	1.03 ± 0.01	1.96 ± 0.01**
4	NK	4.10 ± 0.01	1.21 ± 0.00	2.10 ± 0.01
	SK1	4.16 ± 0.01**	1.22 ± 0.00	1.98 ± 0.01**
	SK2	4.14 ± 0.01	1.20 ± 0.00**	2.11 ± 0.00**
	SK3	3.78 ± 0.01**	1.44 ± 0.01**	1.82 ± 0.01**
	SK4	4.17 ± 0.01	1.25 ± 0.00**	1.98 ± 0.01

Results are expressed as mean ± SD. *p < 0.05 and **p < 0.01, vs. NK (kimchi without LAB).
